# Gyroscopic Stereotactic Radiosurgery: A Retrospective Analysis

**DOI:** 10.7759/cureus.88553

**Published:** 2025-07-22

**Authors:** Dylan R Conroy, Susan M Woody, Ying E Niu, Yuan E Yuan, Keith Unger, Andrew A Satinsky, Amjad Anaizi, Matthew E Witek, Dalong Pang

**Affiliations:** 1 Radiation Oncology, MedStar Georgetown University Hospital, Washington, DC, USA; 2 Neurosurgery, MedStar Georgetown University Hospital, Washington, DC, USA

**Keywords:** brain stereotactic radiosurgery, radio-neurosurgery, stereotactic srs, zap, zap-x radiosurgical system

## Abstract

Introduction

Stereotactic radiosurgery (SRS) is a safe and effective non-invasive treatment for both primary and secondary brain tumors. Recently, a gyroscopic frameless, self-contained, and self-shielded radiosurgery system has been clinically implemented. The device is equipped with a 2.7-MV linear accelerator, capable of delivering up to 1500 MU/minute, and onboard image guidance for initial setup and tracking. Herein, we present our institution’s initial experience with this novel system.

Methodology

Between September 2020 and July 2023, 30 patients (77%) with brain metastases, 8 patients (20.5%) with benign skull base tumors, and 1 patient (2.5%) with an arteriovenous malformation were treated for a total of 107 lesions (1-11 lesions per patient) using 243 radiation fractions, with each patient receiving between 1 and 5 fractions.

Results

The median delivered fractional dose was 1350 cGy (range, 500-2,400 cGy) in 3 fractions (range, 1-5) for a total dose of 2,400 cGy (range, 1,200-3,500 cGy) prescribed to the 63% isodose line (range, 50%-85%). The median lesion volume was 1.1 cc (range, 0.04-28.04 cc). The median parametric values of conformity, homogeneity, and dose falloff were calculated: confidence interval (CI) = 1.43 (1.21-1.86), homogeneity index (HI) = 1.52 (1.14-2.00), and gradient index 50 (GI50) = 3.16 (2.49-4.88). Median treatment time was 24 minutes per lesion (range, 9-123 minutes), and median isocenters per lesion was 4 (range, 1-15), with a median of 74 beams per lesion delivered (range, 23-195). Acute toxicities were limited to 6 (16.2%) patients. Late toxicity was limited to seven lesions, causing radiation necrosis, five symptomatic, and one patient with a case of Bell’s palsy. No grade 4+ toxicity was observed. At a median follow-up of 14.7 months (range, 2.4-46.3), local control was 95%.

Conclusions

This is among the largest gyroscopic radiosurgery clinical experiences published to date. The results show that local control and toxicity results align with historical control data for patients treated with other SRS modalities, demonstrating the functionality, performance efficiency, and dosimetric benefits of this new radiosurgery platform.

## Introduction

Stereotactic radiosurgery (SRS), first described by Dr. Lars Leksell in 1951, was envisioned to treat lesions deep within the brain, previously considered inaccessible by standard surgical approaches without significant morbidity [1]. His concept first materialized as an orthovoltage X-ray tube combined with a rigid stereotactic frame. This was later replaced by a cyclotron, and eventually by stationary Cobalt-60 sources [2]. The resulting device, known as the Leksell Gamma Knife®, was first used in 1967 to treat patients with trigeminal neuralgia and obsessive-compulsive disorder [3].

Over the ensuing decades, SRS has proven to be an invaluable instrument to both the neurosurgeon and radiation oncologist. As the first method of dose escalation with both an ablative and cytotoxic biological effect, SRS has established itself as a preferred tool for the treatment of both benign and malignant tumors of the head and neck [4].

Contemporary SRS is characterized by highly conformal ionizing radiation with a steep dose fall off to spare surrounding normal tissue. As opposed to the original Cartesian coordinate system and plain radiographs used in early stereotaxy, modern techniques utilize high-resolution magnetic resonance or computed tomography imaging for targeting. Given the inherent precision and accuracy of SRS, either stereotactic frames or custom-molded face masks are necessary to ensure rigid immobilization during treatment.

Today, SRS can be delivered via a number of devices, including dedicated radiosurgical systems like the Gamma Knife (Elekta, Stockholm, Sweden), CyberKnife (Accuray, Sunnyvale, CA), and Novalis (Brainlab, Feldkirchen, Germany), or multi-use linear accelerators (LINACs).

Recently, the ZAP-X ® Gyroscopic Radiosurgery platform (ZAP Surgical Systems, Inc., San Carlos, CA) became available. This device is a novel, frameless, self-contained, and self-shielding system intended for the radiosurgical treatment of intracranial and cervical spinal lesions [5]. Ionizing radiotherapy is provided by a 2.7-MV S-band linear accelerator mounted within a combination of yoked gimbals, all rotating about a common isocenter [5]. Its integrated self-shielding design precludes the need for a typical, which significantly reduces the cost for its implementation [6].

Herein, we present a retrospective study reporting our institution’s initial experience with the ZAP-X® Gyroscopic radiosurgical system. The objectives of this study were to demonstrate the applicability, dosimetric acceptability, and clinical outcomes of a newly designed, dedicated, vault-free radiosurgical system suitable for use in community settings where shielding infrastructure may be limited. 

## Materials and methods

Study design and participants

This clinical retrospective chart review was approved by the Georgetown-MedStar Institutional Review Board (IRB STUDY00004335). Our analysis included 39 patients with primary or secondary intracranial or base of skull tumors treated with ZAP-X radiosurgery between September 1, 2020, and July 31, 2023. Patients who underwent SRS with or without a prior surgical resection, and had at least a year of follow up were included. We included all patients to correlate with what may be expected at other community centers. If a patient passed away during that year's follow up they were included. All patients were treated within a multidisciplinary team of radiation oncologists, neurosurgeons, and medical physicists.

Treatment planning and delivery

Each patient underwent contrast-enhanced magnetic resonance imaging (MRI) or high-resolution computed tomography of the brain before treatment for diagnosis. Treatment simulation entailed the creation of a custom-molded thermoplastic facemask for immobilization and reproducibility. A thin-cut (1.25 mm) contrast-enhanced treatment planning CT scan of the head and neck was obtained. The planning CT scans and pretreatment thin-cut T1 post-contrast (MPRAGE sequence) MRIs were fused in the treatment planning software and used for target volume delineation. Lesions were contoured on all visualized axial slices of the planning CT, with MRI used for verification. For postoperative cavities and in select intact lesions, a 1-2 mm volumetric expansion was applied.

Treatment planning was conducted using the ZAP-X Treatment Planning System (TPS), an isocenter-based platform capable of both forward and inverse planning, utilizing sphere-packing and beam-weighting techniques. The prescription isodose was determined by the treating radiation oncologist and medical physicist, taking into account the target volume size, tumor histology, proximity to adjacent critical structures, and history of prior intracranial radiation.

Patients were treated in the supine position. SRS was delivered in 1-5 fractions. Fractionated SRS (fSRS) was performed every other day. In patients undergoing fSRS, treatment length was calculated on the basis of the total number of elapsed days from the first to last treatment fraction.

Outcomes assessment

Patients were monitored through routine clinical follow-up and evaluated for overall survival and treatment-related toxicity. Contrast-enhanced MRI or CT scans were obtained every three months for metastatic lesions and six months for benign lesions to assess response, including survival, recurrence, and adverse events. Patients were evaluated for adverse events, which were graded according to the Common Terminology Criteria for Adverse Events (CTCAE v5.0) and recorded by the treating radiation oncologists.

Statistical analysis

Statistical analysis was performed using SPSS Statistics (IBM Corp., Armonk, NY) to calculate the mean time to progression or death. The follow-up duration was defined as the time from the date of treatment completion to the date of most recent imaging, clinical follow-up, or date of death. Local control was defined as the lack of radiographic or clinical progression. The Kaplan-Meier method was used to calculate the probability of overall survival and local control.

## Results

Patient and lesion characteristics 

Thirty-nine patients were identified as having undergone ZAP-X radiosurgery with a total of 107 lesions (range, 1-11 lesions per patient) treated in 243 fractions (Table 1). Twenty-three (59%) were female, and 16 (41%) were male. The median age at the time of treatment was 63 (range, 25-86) years. Patients were fit overall, with a median Eastern Cooperative Oncology Group (ECOG) performance status of 2 (range, 0-3). Primary tumor histologies and treatment sites were diverse (Table 2).

**Table 1:** Patient characteristics. Patient characteristics, including age, gender, ECOG performance status, and histology of primary tumor. NSCLC, non-small cell lung cancer; SCLC, small cell lung cancer

Patient characteristics	Number (*n* = 39)	Range/Percentage (*n* = 39)
Age (Median, years)	63 years	Range: 25-86
Female	23	59
Male	16	41
ECOG performance status (Median)	2.0 PS	Range: 0-3
NSCLC primary	10	25.6
Breast primary	8	20.5
SCLC primary	3	7.7
Renal cell carcinoma primary	3	7.7
Melanoma primary	2	5.1
Bladder cancer primary	2	5.1
Colorectal Cancer Primary	2	5.1
Vestibular schwannoma	4	10.3
Meningioma	3	7.7
Glomus jugulare paraganglioma	1	2.6
Arteriovenous malformation	1	2.6

**Table 2:** A summary of lesion characteristics. Lesion characteristics included malignancy status (benign or malignant), histology, anatomical location, and whether the lesion was intact or postoperative. NSCLC, non-small cell lung cancer; SCLC, small cell lung cancer

Lesion summary	Number of lesions (*n* = 107)	Percentage
Malignant	98	92%
Benign	9	8%
NSCLC primary	42	39.3%
Breast primary	19	17.8%
SCLC primary	14	13.1%
Renal cell carcinoma primary	9	8.4%
Melanoma primary	2	1.9%
Bladder cancer primary	9	8.4%
Colorectal cancer primary	3	2.8%
Vestibular schwannoma	4	3.7%
Meningioma	3	2.8%
Glomus jugulare paraganglioma	1	0.9%
Arteriovenous malformation	1	0.9%
Frontal lobe	22	20.6%
Parietal lobe	30	28%
Occipital lobe	8	7.5%
Temporal lobe	15	14%
Cerebellum	22	20.6%
Internal auditory canal	4	3.7%
Jugular foramen	1	0.9%
Brainstem	1	0.9%
Planum sphenoidale	1	0.9%
Corpus callosum	1	0.9%
Pineal	1	0.9%
Putamen	1	0.9%
Intact	95	89%
Postoperative	12	11%

Treatment characteristics 

The median delivered fractional dose was 1,350 cGy over 3 fractions, for a total dose of 2,400 cGy, prescribed to the 63% isodose line. More detailed treatment characteristics are available in Table 3.

**Table 3:** Treatment characteristics. GTV, gross tumor volume; PTV, planning target volume

Dosimetry parameters	Median/Number	Range/Percentage
Lesion volume (cc)	1.1	0.04-28.04
Fractional dose (cGy)	1350	500-2400
Total dose (cGy)	2400	1200-3500
Prescription isodose line (%)	63	50-85
Beams per lesion	74	23-195
Isocenters per lesion	4	1-15
Treatment time (minutes per lesion)	24	9-123
Conformality index (CI)	1.43	1.21-1.86
Homogeneity index (HI)	1.52	1.14-2.00
Gradient index 50 (GI50)	3.16	2.49-4.88
Gradient index 25 (GI25)	9.99	6.33-19.00
Brain V12 (cc)	12.10	0-121.9
Brain V10 (cc)	15.36	0-166.70
Brain Dmean (cGy)	121.60	4.9-552.3
Lesions treated with 1 fraction; *n* = 107	46	43.0%
Lesions treated with 3 fractions; *n* = 107	54	50.5%
Lesions treated with 5 fractions; *n* = 107	7	6.5%
0 mm GTV to PTV margin; *n* = 107	58	54.2%
1 mm GTV to PTV margin; *n* = 107	6	5.6%
2 mm GTV to PTV margin; *n* = 107	42	39.3%
3 mm GTV to PTV margin; *n* = 107	1	0.9%

**Figure 1:**
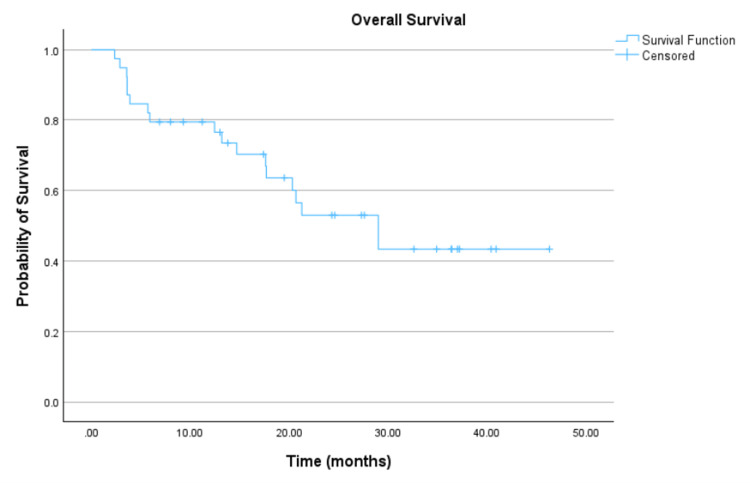
Overall survival.

**Figure 2:**
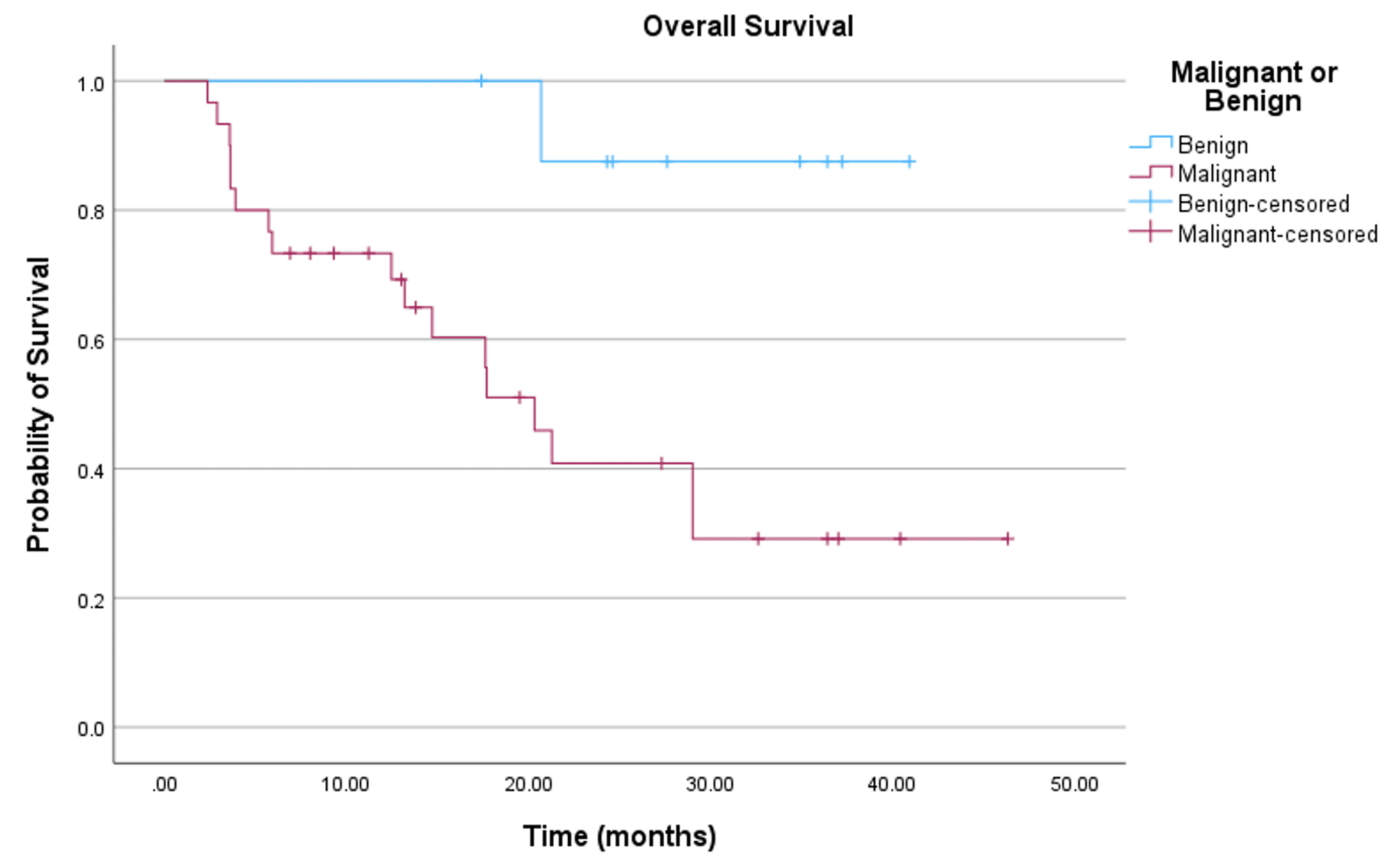
Overall survival (malignant vs. benign).

The median follow-up per lesion was 14.1 months (range, 1.5-46.3). Eighteen (46.2%) patients died within the study interval. Kaplan-Meier analyses were performed, as shown in Figure 1 and Table 4. The median overall survival for the entire cohort was estimated at 29.0 months (95% CI: 17.2-40.7). For the malignant and benign histology subgroups, the median overall survival was estimated at 20.3 months and was not reached, respectively, as shown in Figure 2 and Table 4. 

**Table 4:** Survival/recurrence outcomes. CI, confidence interval

Outcome measure	Time (months)
Median survival (95% CI) (*n* = 39)	29.0 months (17.2-40.7)
Mean survival (*n* = 39)	28.3 months (22.4 – 34.2)
Malignant histology (median) (*n* ​​​​​​​ = 39)	20.3 months
Benign histology (median) (*n* ​​​​​​​ = 39)	Not met
Local control mean (95% CI) (*n* ​​​​​​​ = 107)	43.201 months (40.7-45.7)
Median time to recurrence (range)	12.8 months (0.5-34.0)

**Figure 3:**
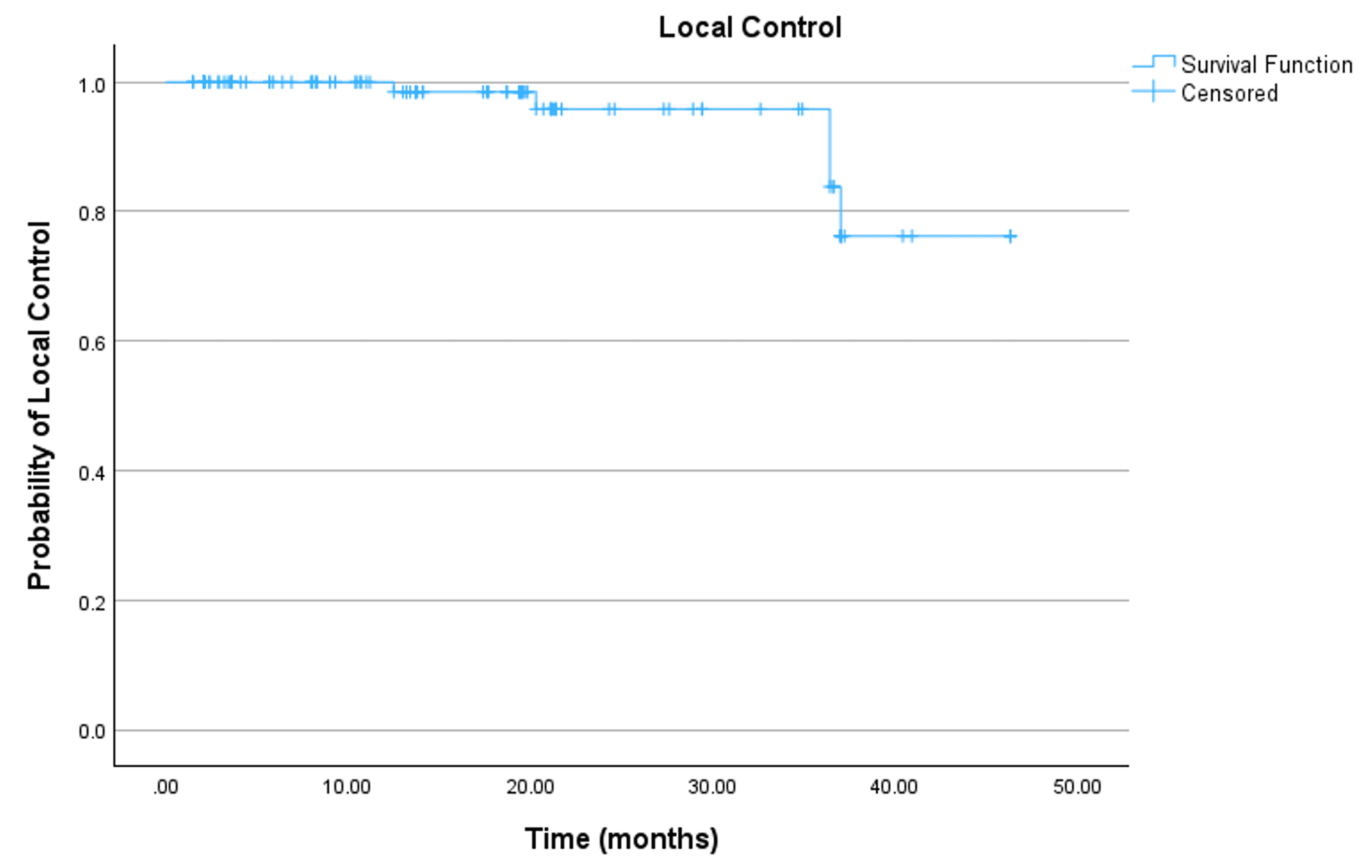
Local control.

Of the 107 lesions, five (4.7%) demonstrated local recurrence/progression. Median local control was not reached. The mean local control duration was estimated to be 43.201 months (95% CI: 40.730-45.672). One- and three-year local control was estimated to be 98.5% and 83.8%, respectively (Figure 3; Table 4). The histologies of the five recurrent lesions were small cell lung cancer (SCLC), renal cell carcinoma (RCC), RCC, RCC, and breast cancer. These lesions recurred at 10.5, 0.5, 34.0, 34.0, and 12.8 months, respectively.

Acute and late toxicities were recorded and graded according to CTCAE v5.0 and are given in Table 5. Acute toxicity was limited to 6 (15.4%) patients - one patient with a meningioma experienced CTCAE grade 1 radiation dermatitis. One patient with brain metastases from metastatic breast cancer experienced transient CTCAE grade 1 nausea/vomiting after her second fraction. One patient with brain metastases from metastatic renal cell carcinoma experienced worsening of pre-existing left-sided weakness, which resolved with steroids (CTCAE grade 2). One patient with brain metastases from metastatic NSCLC and a history of epilepsy experienced a seizure after her first fraction, which required hospitalization (CTCAE grade 3). One patient with brain metastases from metastatic breast cancer experienced new-onset headaches, which resolved with steroids (CTCAE grade 2). Finally, one patient with brain metastases from metastatic NSCLC developed worsening pre-existing ataxia, which resolved with steroids (CTCAE grade 2).

**Table 5:** Toxicity profile.

Toxicity	Number (%)
CTCAE grade 1 acute toxicity (*n* =39)	2 (5.4%)
CTCAE grade 2 acute toxicity (*n* =39)	3 (8.1%)
CTCAE grade 3 acute toxicity (*n* =39)	1 (2.7%)
CTCAE grade 4 acute toxicity (*n* =39)	0 (0%)
CTCAE grade 5 acute toxicity (*n* =39)	0 (0%)
CTCAE grade 1 late toxicity (*n* =39)	0 (0%)
CTCAE grade 2 late toxicity (*n* =39)	3 (8.1%)
CTCAE grade 3 late toxicity (*n* =39)	1 (2.7%)
CTCAE grade 4 late toxicity (*n* =39)	0 (0%
CTCAE grade 5 late toxicity (*n* =39)	0 (0%)
Symptomatic radiation necrosis (*n* = 107)	5 (4.7%)
Asymptomatic (radiographic finding) radiation necrosis (*n* = 107)	2 (1.9%)
Total radiation necrosis (*n* = 107)	7 (6.5%)

Symptomatic radiation necrosis (RN), as reviewed by a multidisciplinary committee, and follow-up imaging was limited to 3 (7.7%) patients, corresponding to 5 lesions (4.7%). RN symptoms resolved with a course of dexamethasone (CTCAE grade 2). One patient with a left-sided vestibular schwannoma developed left-sided Bell’s palsy seven months after undergoing treatment. This did not resolve after treatment with prednisone and acyclovir (CTCAE grade 3). Two patients (5.1%) developed asymptomatic RN, in two lesions (1.9%) as identified on surveillance imaging. One patient was suspected to have asymptomatic RN, but was later noted to have suspected progression. A summary of the clinical outcomes can be found in Table 4.

Case presentations

Patient 1

**Figure 4:**
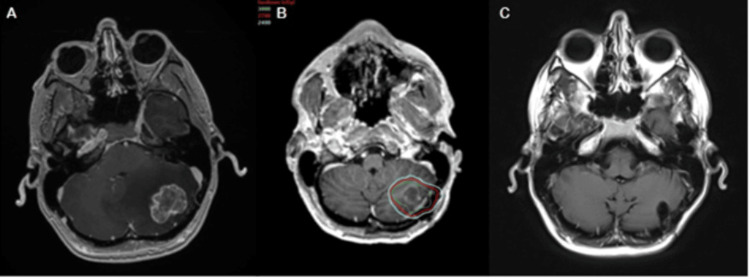
A 42-year-old patient with metastatic colorectal cancer (CRC). (A) Preoperative MRI. (B) Postoperative MRI with overlaid radiosurgical treatment plan. Representative isodose lines: cyan (2,400 cGy), red (2,700 cGy), and green (3,000 cGy). fSRS was prescribed to 2,700 cGy in 3 fractions to the 67% isodose line. (C) MRI performed 15 months post-treatment.

A 42-year-old male with colorectal cancer presented with metastasis to the cerebellum and subsequently underwent primary resection. Figure 4A shows a preoperative MRI demonstrating a 3.4 cm lesion in the left cerebellum with surrounding vasogenic edema, and Figure 4B shows a postoperative MRI with the overlaid radiosurgical treatment plan. Representative isodose lines: cyan (2,400 cGy), red (2,700 cGy), and green (3,000 cGy). fSRS was prescribed to 2,700 cGy in 3 fractions, corresponding to the 67% isodose line. MRI was performed 15 months post-treatment with no evidence of recurrence (Figure 4C).

Patient 2

**Figure 5:**
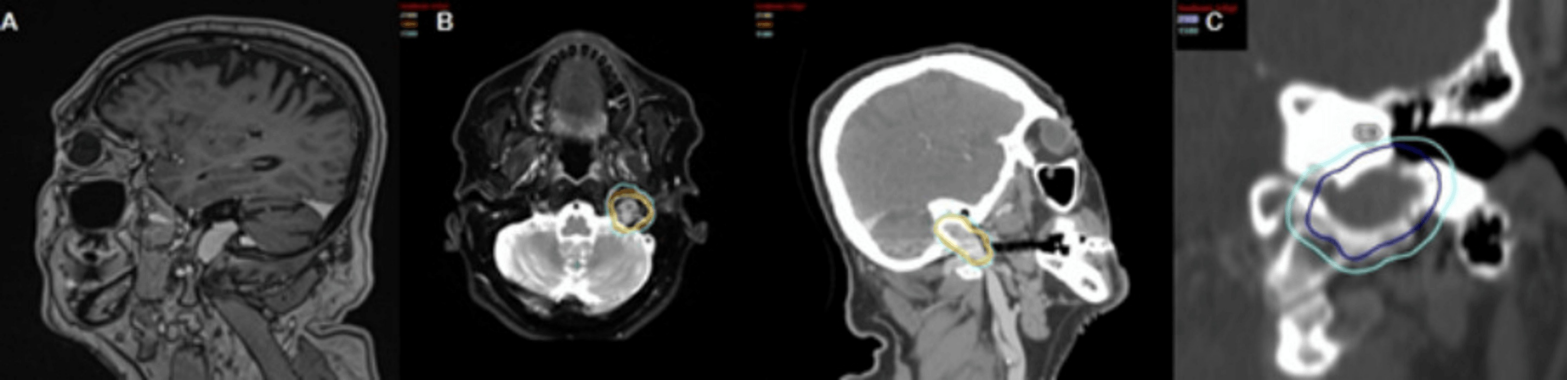
Sixty-seven-year-old male with left-sided glomus jugulare paraganglioma (A) Diagnostic MRI. (B) Overlaid radiosurgical treatment plan. Representative isodose lines: yellow (2,100 cGy), orange (1,800 cGy), and cyan (1,500 cGy). (C) Close-up of the left cochlea, which received a Dmax of 1300 cGy in 3 fractions.

A 67-year-old male presented with a left-sided glomus jugulare paraganglioma. Figure 5A shows diagnostic MRI demonstrating a 2.5 cm enhancing lesion within the left jugular fossa, consistent with a glomus jugulare paraganglioma; Figure 5A shows the overlaid radiosurgical treatment plan. Representative isodose lines: yellow (2,100 cGy), orange (1,800 cGy), and cyan (1,500 cGy). fSRS was prescribed to 2,100 cGy in 3 fractions to the 65% isodose line. Figure 5C shows a close-up of the left cochlea, which received a Dmax of 1300 cGy in 3 fractions.

## Discussion

While first devised for use in functional neurosurgery, radiosurgery has grown to both augment and replace open neurosurgical procedures in the fields of tumor, stereotactic/functional, vascular, and spinal neurosurgery [1].

As reflected in this cohort of patients, the most common indication for SRS is the treatment of intracranial metastases [7]. An estimated 20%-40% of cancer patients will develop intracranial metastases throughout their disease [8,9]. Historically, these have been treated with neurosurgical resection and/or whole-brain radiation therapy (WBRT) [9,10]. While effective for local control, WBRT has a well-established side effect profile, including significant neurocognitive decline [11]. Beginning in the 1990s, studies investigated the use of SRS combined with WBRT and eventually SRS alone versus WBRT for one to three brain metastases, demonstrating comparable local control and overall survival with a significant reduction in toxicity [11-15]. More recently, concrete phase III data have been published supporting the use of SRS for up to 10 brain metastases, with one-year local control rates approaching and exceeding 90% [16-19].

Outside of intracranial metastases, radiosurgery continues to be deployed in the treatment of functional and vascular disorders. The debilitating pain of trigeminal neuralgia can be effectively relieved with SRS, which has remained a preferred treatment modality given its efficacy and low complication rate [20-23]. Patients with vestibular schwannomas have a non-invasive alternative to surgery, with the potential for robust local control and hearing preservation [24-28]. And arteriovenous malformations can be obliterated in up to 90% of appropriately selected patients at five years after treatment [29-32].

This immense capability and versatility of SRS have led to a paradigm shift in the treatment of intracranial disorders. Since 2003, SRS has been performed more frequently than open craniotomy for the treatment of non-meningioma tumors [33]. As the field has evolved into a clinical staple, a heightened emphasis on patient experience and ease of use has emerged.

SRS has historically relied upon the fixation of an external frame to the patient’s skull to accomplish its requisite rigid immobilization. With both Gamma Knife systems and early linear accelerator platforms, this was achieved through the use of invasive skull pins - a practice that can cause considerable discomfort during frame fixation and throughout treatment [34,35]. Aside from the obvious drawbacks to this approach, it also often precludes or discourages the use of fractionated radiosurgery, which has demonstrated a favorable local control profile with a reduction in late RN [16, 36]. Thus, in the late 1990s, several *frameless* stereotactic systems were developed, utilizing custom-fit thermoplastic masks for immobilization and reproducibility. These frameless systems have demonstrated equivalent clinical outcomes to frame-based systems while maintaining submillimeter accuracy, all with significant advantages in patient comfort and the ability to fractionate [37-39]. This accuracy is further augmented with available image-guided and image-tracking systems in modern radiosurgical systems, such as image-guided radiotherapy (IGRT), cone-beam CT, fiducial tracking, and cranial tracking [38,40-44].

The ZAP-X emerged from this era of significant technological growth as a dedicated, self-contained, and self-shielded radiosurgery system. Technical reports are available detailing the novel components of the machine, which are summarized in the Technical details section [5,6,45-49].

In terms of toxicity, RN is generally calculated per lesion and is highly variable in reports, from 0 to 30% due to the factors affecting the risk of RN, including radiation dose and volume, systemic therapy, and tumor histology [50]. Some recent data suggest values of about 4% for Gamma Knife Radiosurgery (GKRS) and LINAC-based SRS [51] compared to our 6.5%. Of note, one patient had three lesions of RN, out of 11 total lesions treated over one year. Elimination of this patient provides a total RN rate of 3.7%, more in line with the aforementioned reports. Overall, a larger patient population with more treated lesions is needed to further elucidate details.

Technical details

Gyroscopic Gimbals

The principal design of the ZAP-X is akin to a gyroscope. A combination of yoked gimbals rotates about a common isocenter. The majority of the devices' integral components are mounted on or within the rotating spherical patient treatment chamber, including the LINAC, radiofrequency power source, waveguide, shielding, kV imager, and MV detector [5]. This construct allows the beam to cover approximately 2π steradians of solid angle (in comparison to 1.2π steradians for Gamma Knife systems).

Self-Shielding

ZAP-X is a self-shielded system and thus obsolesces the traditional radiation vault [5,6]. The Monte Carlo dose algorithm BEAMnrc was utilized to model the path of both primary radiation, as well as leakage and scatter, to determine the placement, thickness, and composition of shielding materials to be used [6]. The final construction is composed primarily of iron and consists of two shields that move independently on axes separated by an angle of 45 degrees, as well as a pneumatic door on a steel frame [5]. The design ultimately limits the leakage of radiation to the patient to less than 0.1% while keeping exposure to those outside a 1-m perimeter of the machine less than 1 mSv/year [6]. This aligns with the recommended maximum annual radiation dose for non-radiation workers as per the NCRP [52].

The benefits of this novel self-shielding system are multiple: installation of the system does not require the design and construction of radiation bunkers, thus reducing both cost and physical resource expenditure. The calculated yearly exposure above is based upon a heavy workload of 2,250 annual single-isocenter treatments of 20 Gy each and still manages to minimize radiation exposure levels that are not only acceptable to radiation workers (allowable annual dose equivalent 50 mSv) but also to the general public (1 mSv/year) [6,52].

LINAC, Collimator, and Beam Profile

Ionizing radiation is produced by a 2.7-MV S-band linear accelerator mounted to the yoked gimbal apparatus. 2.7-MV was specifically chosen for the treatment of intracranial targets (at ~5 cm depth), as this produces a 66% PDD at this depth, which is optimal for radiosurgery [5,45]. The dose rate can reach 1,500 MU/minute with a source-to-axis distance (SAD) of 45 cm [5].

Beam collimation is accomplished by a novel revolving tungsten collimator wheel with eight different divergent circular paths cut through the wheel, with diameters of 4.0, 5.0, 7.5, 10.0, 12.5, 15.0, 20.0, and 25.0 mm (as measured at machine isocenter) [48]. Currently, only one collimator size can be used per isocenter, and the system can automatically change the collimator during treatment.

The combination of low energy, short SAD, and cylindrical collimation allows for an exceptional penumbra, ideally suited for radiosurgery. For example, the 4.0 mm collimator produces a penumbra of 1.8 mm [48].

The linear accelerator and its resultant beam data have been internally and externally validated [45,46].

Patient Immobilization/Tracking

The patient’s head is immobilized in the treatment position with a frameless, custom-molded thermoplastic mask. Pairs of non-coaxial kilovolt (kV) images are captured via an onboard system, which also rotates about the designated isocenter. Similar to the cranial-tracking ability of CyberKnife, skeletal anatomy serves to align the treatment target with the isocenter. Automatic patient registration is performed before and during treatment [6,53].

Megavoltage Radiation Detector

Ultimately, precise beam delivery must be coupled with accurate dose delivery to ensure the success of radiosurgical treatment. Previous radiosurgical systems are limited in monitoring the dose delivered to the patient. The ZAP-X system addresses this issue with a first-of-its-kind real-time megavoltage (MV) imager. The MV imager is factory calibrated and thus does not require the user to calibrate upon receipt [47].

Built upon the principle that the residual dose from a delivered beam is a function of the initial dose, collimation, and tissue attenuation, this system compares real-time to pre-calculated/expected values during treatment to confirm that treatment is proceeding as planned [47].

While not yet a replacement for standardized quality assurance testing, this system provides an integrated and streamlined augmenter and, thus, an added layer of safety. Future applications could include identifying gross deviations in patient anatomy (e.g., tumor shrinkage, weight loss, patient movement).

Limitations

There are limitations to this retrospective review. While the total number of patients is large in terms of gyroscopic radiosurgery data, the number of patients is still limited. Moreover, the patients were skewed heavily for brain metastases, with less information on benign conditions. Therefore, extrapolation of the effect of gyroscopic surgery for those conditions remains very limited, and conclusions should not be inferred without more information. We are continuing to follow more patients and hope to expand upon these numbers in the future. Also, we were limited to patients who had one year of follow-up, so long-term outcome data is still pending. In addition, while a majority of our lesions are metastatic, some are benign and vascular-based based and this may affect data overall, especially in terms of local control and survivability. Overall, we will continue to follow and expand upon this work to further compare gyroscopic radiosurgery to more readily available radiosurgical methods.

## Conclusions

This is among the largest published clinical experiences to date utilizing the ZAP-X Gyroscopic radiosurgical platform. Given this cohort of 39 patients and modest follow-up, any conclusions about the long-term efficacy of the platform are preliminary. However, the local control rate is comparable to historical radiosurgical data. Additional data on longer term toxicity is needed in order to further elucidate rates of toxicity. As such, additional follow-up is planned for this cohort, and prospective trials comparing ZAP-X SRS to clinical mainstay devices like Gamma Knife, CyberKnife, and LINAC-based SRS are warranted. Overall, this paper demonstrates that ZAP is a self-shielding SRS system that can be used in areas where other forms of SRS may not be feasible due to the need for frame setup or radiation vaulting.
